# MGRL: Predicting Drug-Disease Associations Based on Multi-Graph Representation Learning

**DOI:** 10.3389/fgene.2021.657182

**Published:** 2021-04-08

**Authors:** Bo-Wei Zhao, Zhu-Hong You, Leon Wong, Ping Zhang, Hao-Yuan Li, Lei Wang

**Affiliations:** ^1^The Xinjiang Technical Institute of Physics and Chemistry, Chinese Academy of Sciences, Ürümqi, China; ^2^University of Chinese Academy of Sciences, Beijing, China; ^3^Xinjiang Laboratory of Minority Speech and Language Information Processing, Ürümqi, China; ^4^The School of Computer Sciences, BaoJi University of Arts and Sciences, Baoji, China; ^5^School of Computer Science and Technology, China University of Mining and Technology, Xuzhou, China

**Keywords:** drug, disease, drug repositioning, multi-graph representation learning, graph embedding

## Abstract

Drug repositioning is an application-based solution based on mining existing drugs to find new targets, quickly discovering new drug-disease associations, and reducing the risk of drug discovery in traditional medicine and biology. Therefore, it is of great significance to design a computational model with high efficiency and accuracy. In this paper, we propose a novel computational method MGRL to predict drug-disease associations based on multi-graph representation learning. More specifically, MGRL first uses the graph convolution network to learn the graph representation of drugs and diseases from their self-attributes. Then, the graph embedding algorithm is used to represent the relationships between drugs and diseases. Finally, the two kinds of graph representation learning features were put into the random forest classifier for training. To the best of our knowledge, this is the first work to construct a multi-graph to extract the characteristics of drugs and diseases to predict drug-disease associations. The experiments show that the MGRL can achieve a higher AUC of 0.8506 based on five-fold cross-validation, which is significantly better than other existing methods. Case study results show the reliability of the proposed method, which is of great significance for practical applications.

## Introduction

In recent years, the long hours and high costs of developing new drugs have been significant constraints (DiMasi et al., 2003; Adams and Brantner, 2006). Most new drugs already cost more than billions of dollars to build, and it will take many years to bring them to market (Wei et al., 2019). Unfortunately, as the cost of drug development has risen, drug profits have fallen. Identifying potential drug-disease associations is a top priority in drug discovery, and the side effects of some drugs have been confirmed by clinical observation.

Recently, a large number of computing methods based on drug-disease associations prediction have been proposed (Huang et al., 2013; Li et al., 2016; Zickenrott et al., 2016; Zhang et al., 2017a; Xue et al., 2018; Yella et al., 2018; Cui et al., 2019; Xuan et al., 2019; Chen et al., 2020; Jarada et al., 2020). Gottlieb et al. (2011) proposed the prediction method based on the computational similarity framework between drug-drug similarity and disease-disease similarity and predict unknown correlations by constructing similar characteristics of recently known drug-disease associations. Luo et al. (2018) proposed a drug repositioning recommendation system to predict new drug-disease associations by constructing a heterogeneous drug-disease interactions network. Wang et al. (2014) designed a computing framework based on a heterogeneous network model to calculate the similarity between drug pairs of diseases through heterogeneous graphs of drug-target information. Zhang et al. (2017a) constructed the known drug-disease association into a drug-disease bipartite graph network and proposed a similarity-based graph to predict the new drug-disease associations method. Liang et al. (2017) proposed a new computational method that integrates the chemical, target region, and target labeling information of a drug. Jiang et al. (2020) combined various disease characteristics and drug characteristics and proposed a sparse automatic coder and a rotating forest fusion method for humans.

Most of the existing drugs are used to discover the relationship between potential drugs and diseases by extracting similarities between drugs and diseases (Li and Lu, 2012; Zhang et al., 2014, 2017b, 2018; Luo et al., 2016). Chen et al. (2020) used network embedding and traditional attributes to predict drug targets by integrating the correlation between various molecules. According to research, graph neural network has been widely used in related biological and medical fields (Li et al., 2020; Wang et al., 2020; Yue et al., 2020). Wang et al. (2019) proposed a prediction method for embedding drug-disease associations networks using graph neural networks. Based on the similarity between drugs and diseases, Yu et al. (2020) introduced graph convolutional neural networks to predict potential drug-disease associations. As a result, only a handful of drugs and diseases with rich information can be used for prediction. Therefore, how to solve these challenges is urgent. Inspired by existing research (Guo et al., 2019, 2020; Yi et al., 2019, 2020). We propose a computational method of representation learning based on multi-graph by learning features from local and global perspectives, respectively.

**Figure 1:**
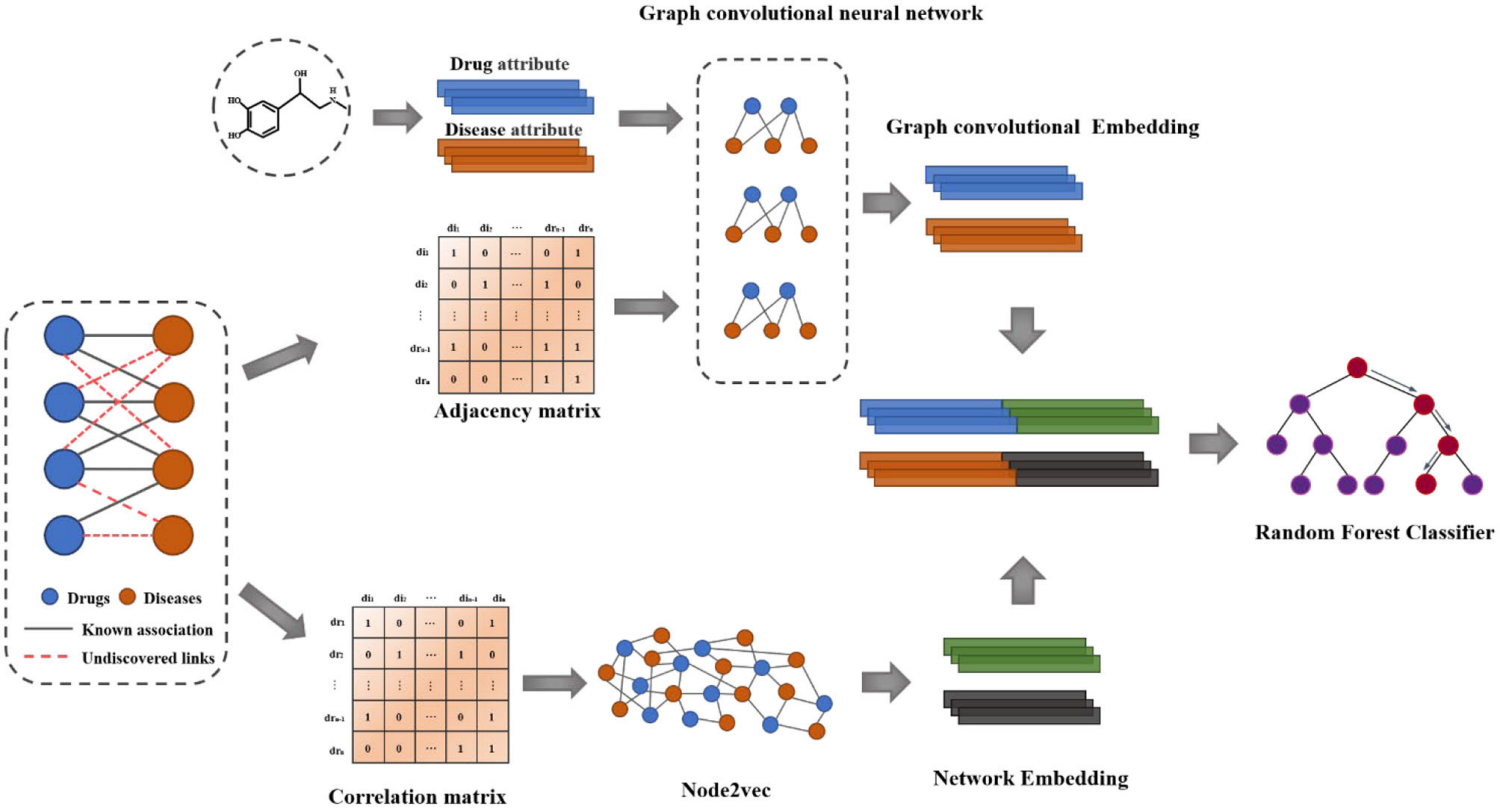
Flowchart of the proposed method.

In this paper, we propose a novel computational model based on Multi-graph representation learning (MGRL) to predict drug-disease associations, which is mainly divided into three parts. First of all, The self-attributes of drugs and diseases are pre-trained by using the graph convolutional neural network to generate the graph convolutional neural network features. Then, node2vec (Grover and Leskovec, 2016) was used for network representation of the drug-disease associations. Finally, the two obtained multi-dimensional information features were combined, and the latent drug-disease associations were predicted using Random Forest Classifier (Amaratunga et al., 2008). The overall workflow of the Multi-graph representation learning (MGRL) is demonstrated in Figure 1. Experiments results show that the MGRL have higher accuracy and AUC for predicting new drug-disease associations and comparing state-of-the-art methods. The case study shows that the model MGRL could better help medical researchers discover new drug-disease associations.

## Materials and Methods

### Datasets

The Comparative Toxicogenomics Database (CTD) (Davis et al., 2017) provides information about the relationship between chemicals and gene products and diseases. Concentrate and combine molecular pathways to uncover real chemicals and understand environmental influences on etiology and disease mechanisms. According to Zhang et al.'s (2018) treatment method of drug-disease associations in CTD, we obtained 18,416 drug-disease relationship pairs. We use the DrugBank (Law et al., 2014) database to obtain the chemical structure of drugs. The database is an open and comprehensive drug resource library, including the chemical structure of drugs, drug targets, various proteases, and so on. The description of the disease information collection from the Medical Subject Headings (MeSH). Therefore, the benchmark dataset contain 18,416 drug-disease pairs, including 269 drugs and 598 diseases.

### Drug Morgan Molecular Fingerprint

In this paper, the simplified molecular-input line input specification (SMILES) is adopted (Weininger, 1988), which describes the chemical structure of drug molecules. The characteristics of chemical molecules are represented by RDkit (Landrum, 2013), a tool kit that can be used to represent chemical information.

### Disease Semantic Description Information

In the experiment, the network descriptors in the MeSH database were used to process the disease data (Wang et al., 2010). The data is downloaded from the National Library of Medicine (http://www.nlm.nih.gov/). The MeSH database provides a strict disease classification system, so it plays an essential role in the study of the attributes of diseases and the relationship between diseases. In general, the MeSH descriptor is described as a directed acyclic graph (DAG) of diseases, where diseases are represented by the nodes. In other word, each disease can be represented as a structure of DAG. For instance, *DAG*_*A*_ = (*A, T*_*A*_, *E*_*A*_), in which the collection of all the ancestor nodes of *A* is represented by *T*_*A*_, including node itself, and *E*_*A*_ is a collection of links to the node. Therefore, by assuming that the contribution of disease *t* to the semantics of disease *a* is *D*(*a*), the following formula can be obtained:

(1) $$\{\begin{array}{l} \;{\text{D}}_{\text{a}}(\text{a})=1\\ {\text{D}}_{\text{a}}(\text{t})=\text{max }\{\mu *{\text{D}}_{\text{a}}({\text{t}}^{\prime })|{\text{t}}^{\prime }\;\in \text{ children of t}\}\text{ if t }\neq \text{ a} \end{array}$$

where μ is the semantic contribution factor of the connection edge *E*(*T*) between the parent node *T* and the child node *t*. Therefore, the semantic value of disease can be defined as:

(2) $$\begin{array}{r} \text{DV}\left(\text{a}\right)=\sum_{\text{t}\in {\text{T}}_{\text{a}}}{\text{D}}_{\text{a}}\left(\text{t}\right) \end{array}$$

In conclusion, a measure of semantic similarity between the two diseases can be calculated by their relative locations. The formula is as follows:

(3) $$\text{Simlarity}({\text{D}}_{\text{a}},{\text{D}}_{\text{b}})=\frac{\sum_{\text{t}\in {\text{D}}_{\text{a}}\cap {\text{D}}_{\text{b}}}\left[{\text{D}}_{\text{a}}\left(\text{t}\right)+{\text{D}}_{\text{b}}\left(\text{t}\right)\right)]}{\text{DV}(\text{a})+\text{DV}(\text{b})}$$

where *D*_*a*_(*t*) and *D*_*b*_(*t*) are the semantic values of disease *t* related to disease *a* and disease *b*, respectively.

### Graph Convolutional Neural Network

Graph convolutional neural network (GCN) (Kipf and Welling, 2016) is considered as a graph-based semi-supervised learning method for node classification. GCN directly encodes the graph structure by using the neural network model and learns from the supervised target of labeled nodes. Its essence is the first-order local approximation of spectral convolution.

In this work, we consider the multi-layer graph convolutional network as follows:

(4) $$\begin{array}{r} {\text{H}}^{\left(\text{l}+1\right)}=\sigma \left({\tilde{\text{D}}}^{-0.5}\tilde{\text{A}}{\tilde{\text{D}}}^{-0.5}{\text{H}}^{\left(\text{l}\right)}{\text{W}}^{\left(\text{l}\right)}\right) \end{array}$$

where *H* is the network input of layer *l* (initialized input *H* = *X*), $\tilde{D}$ is degree matrix of Ã. Ã = *A* + *I* is the adjacency matrix added to the self-loop, *W* is the weight of training in the neural network, σ is the activation function, and the ReLU function is used.

The traditional graph convolutional neural network is an end-to-end system. How to use it to train the attributes of nodes and get the attributes of nodes after training is the core of the problem we need to solve. Therefore, we have designed a unique graph convolutional neural network. Specifically, let us assume given an adjacency matrix *A*_*n* × *n*_, where *n* represents all nodes (including drugs and diseases), Ã = *A* + *I*, where

(5) $$\begin{array}{r} {\tilde{\text{A}}}_{\text{n}\times \text{n}}=\left[\begin{array}{cccc} 1 & 0 & \cdots & 1\\ 0 & 1 & \cdots & 0\\ \vdots & \vdots & \vdots & \vdots \\ 0 & 0 & \cdots & 1 \end{array}\right]\; \end{array}$$

*I* is a unit matrix of size *n* × *n*. Then, define the attribute of the node as $X_{nk}={\left[x_{1},x_{2},x_{3},\cdots \;,x_{nk}\right]}^{T}$ in which *k* is the attribute dimension of all nodes. Finally, the weight *W*_*k* × *m*_ is initialized randomly, and *m* is equal to 64. The following formula can be obtained:

(6) $$\begin{array}{r} \text{H }=\;\sigma \left(\tilde{\text{A}}\text{XW}\right) \end{array}$$

We used this simplified definition of graph convolution in this work.

### Node2vec

Node2vec (Grover and Leskovec, 2016) is a method that can learn the continuous feature representation of each node in the network. It can map the node to low-dimensional feature space and preserve the network neighborhood of the node to the maximum. Node2vec provides a biased random walk method to obtain the nearest neighbor sequence of vertices, effectively combining DFS (Depth First Search) and BFS (Breath First Search). We assume that node *v* is the current vertex, then the probability of accessing the next vertex *x* is:

(7) $$\text{P}({\text{c}}_{\text{i}}=\text{x}|{\text{c}}_{\text{i-1}}=\text{v})=\{\begin{array}{c} \frac{\pi_{\text{vx}}}{\text{Z}}\text{ if }\left(\text{v},\text{x}\right)\in \text{ E}\\ \text{0 otherwise} \end{array}$$

where π is a vertex *v* and not normalized transition probability between *x*, *Z* is a normalized constant. *c* is the node in the walk and initial *c* = *u*.

Consequently, two super parameters *p* and *q* are introduced to control the strategy of the random walk. It is assumed that the current random walk reaches the vertex *v* after passing the edge (*t, v*). Here, the unnormalized transition probability is set as π_*vx*_ = α_*pq*_(*t, x*)·*w*_*vx*_, where:

(8) $$\alpha_{\text{pq}}\left(\text{t},\text{x}\right)=\{\begin{array}{c} \frac{1}{\text{p}}\text{ if }{\text{d}}_{\text{tx}}=0\\ 1\text{ if }{\text{d}}_{\text{tx}}=1\\ \frac{1}{\text{q}}\text{ if }{\text{d}}_{\text{tx}}=2 \end{array}$$

which *w* is the weight of the edge between the vertices *v* and *x*, *d* is the shortest path distance between vertex *t* and vertex *x*.

## Results

### Five-Fold Cross-Validation

Cross-validation has absolute authority in evaluating the predictive performance of the model, especially for assessing the performance of the model with completed training on new data, which can better solve the problem of model overfitting. In the experiment, we choose five-fold cross-validation. Besides, we choose other evaluation criteria, including accuracy (Acc.), sensitivity (Sen.), specificity (Spec.), precision (Perc.), and Matthews correlation coefficient (MCC). TN, TP, FN and FP are represented as true negatives, true positives, false negatives and false positives. These evaluation indexes are calculated as follows:

(9) $$\begin{array}{r} \text{Acc}=\frac{\text{TN}+\text{TP}}{\text{TN}+\text{TP}+\text{FN}+\text{FP}} \end{array}$$

(10) $$\begin{array}{r} \text{Sen}=\frac{\text{TP}}{\text{TP}+\text{FN}} \end{array}$$

(11) $$\begin{array}{r} \text{Spec}=\frac{\text{TN}}{\text{TN}+\text{FP}} \end{array}$$

(12) $$\begin{array}{r} \text{Prec}=\frac{\text{TP}}{\text{TP}+\text{FP}} \end{array}$$

(13) $$\begin{array}{r} \text{MCC}=\frac{\text{TP}\times \text{TN}-\text{FP}\times \text{FN}}{\sqrt{\left(\text{TP}+\text{FP}\right)\times \left(\text{TP}+\text{FN}\right)\times \left(\text{TN}+\text{FP}\right)\times \left(\text{TN}+\text{FN}\right)}} \end{array}$$

**Figure 2:**
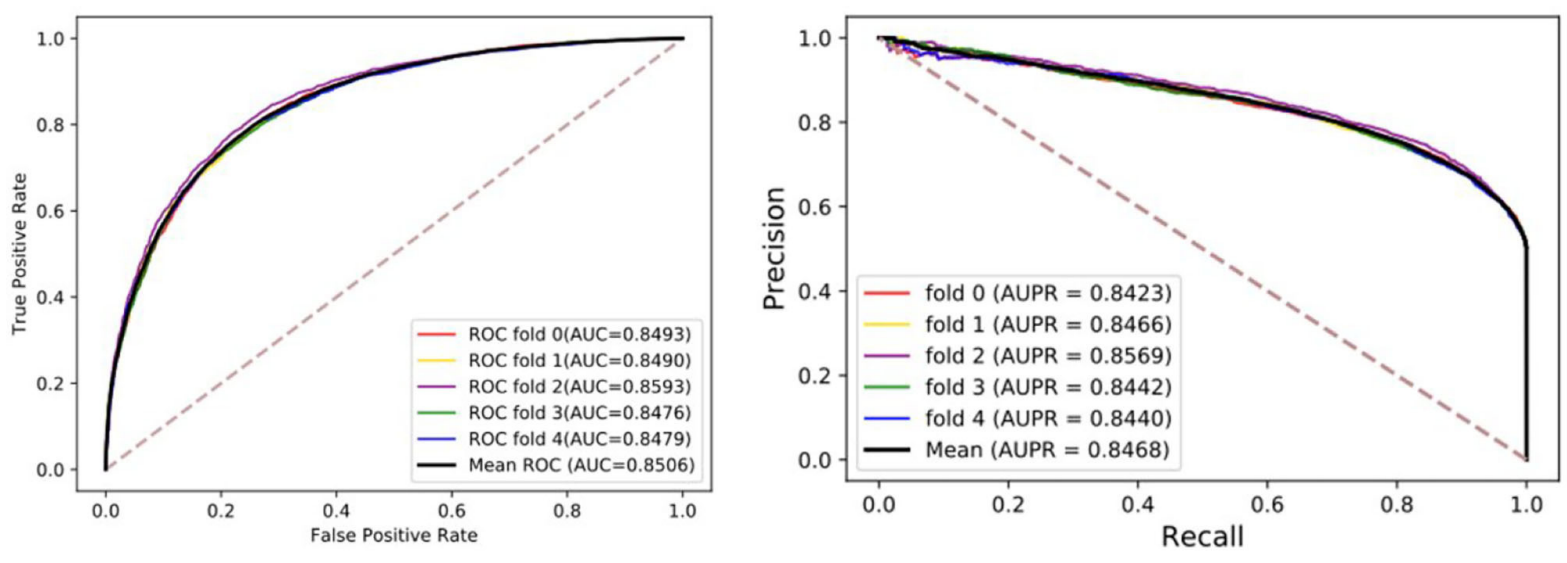
The ROCs, AUCs, PRs, and AUPRs of MGRL under five-fold cross-validation on the benchmark dataset.

To visualize, the ROC curve (receiver operating characteristics) was used to assess our method. The appropriate right approach to the ROC curve should be close to the unit square in the upper left corner. If the ROC curve follows a diagonal line of negative classifiers and connecting identifier points, the predictive effect of random guesses on classifiers is also lacking. AUC was used as an evaluation index, which is the area under the ROC curve. The higher the value, the higher the accuracy. Moreover, the precision-recall diagram (PR) was added to evaluate our model, where AUPR is the area under the PR curve, which can directly reflect the recall rate and accuracy of learners in the whole sample and prevent errors caused by the small number of positive samples. Although the benchmark dataset is stable, we still hope that these evaluation indexes can provide references for the later models. The details of results under five-fold cross-validation are shown in the Table 1 and Figure 2. Through the analysis, it is clear that the MGRL results are outstanding. AUC, AUPR, and various evaluation indexes illustrate that the proposed model has excellent predictive ability.

**Table 1:** Five-fold cross-validation results performed by MGRL.

**Fold**	**Acc. (%)**	**Sen. (%)**	**Spec. (%)**	**Prec. (%)**	**MCC (%)**	**AUC (%)**
0	76.93	73.59	80.27	78.85	53.97	84.93
1	76.47	73.45	79.48	78.16	53.03	84.90
2	77.67	75.11	80.24	79.17	55.42	85.93
3	76.53	74.10	78.96	77.89	53.13	84.79
4	76.70	73.05	80.35	78.80	53.54	84.79
Average	**76.86** **±** **0.49**	**73.86** **±** **0.79**	**79.86** **±** **0.61**	**78.57** **±** **0.53**	**53.82** **±** **0.97**	**85.06** **±** **0.49**

### Evaluate the Impact of Different Feature

**Figure 3:**
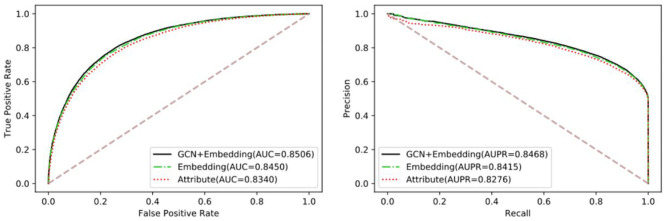
The comparison of different feature using Gradient Boosting Decision Tree classifier.

To verify the performance differences between different features and the advantages of the proposed method, we compared three targeted features, including Attribute, Embedding, and GCN+Embedding. Table 2 and Figure 3 show the benefits of the proposed method under different evaluation indexes. The comparison experiment shows the performance of different features. The attribute performance of the node is the weakest, possibly because the attribute is relatively single. The establishment of multi-graph for node feature extraction has a decisive advantage.

**Table 2:** Comparison of different feature using Random Forest Classifier under five-fold cross-validation.

**Feature**	**Acc. (%)**	**Sen. (%)**	**Spec. (%)**	**Prec. (%)**	**MCC (%)**	**AUC (%)**
Attribute	75.53 ± 0.37	76.38 ± 0.82	74.68 ± 0.47	75.10 ± 0.31	51.07 ± 0.73	83.40 ± 0.45
Embedding	76.31 ± 0.52	72.05 ± 0.64	80.58 ± 0.76	78.77 ± 0.68	52.82 ± 1.06	84.50 ± 0.54
GCN+Embedding	**76.86** **±** **0.49**	**73.86** **±** **0.79**	**79.86** **±** **0.61**	**78.57** **±** **0.53**	**53.82** **±** **0.97**	**85.06** **±** **0.49**

### Comparison With Different Classifiers

**Figure 4:**
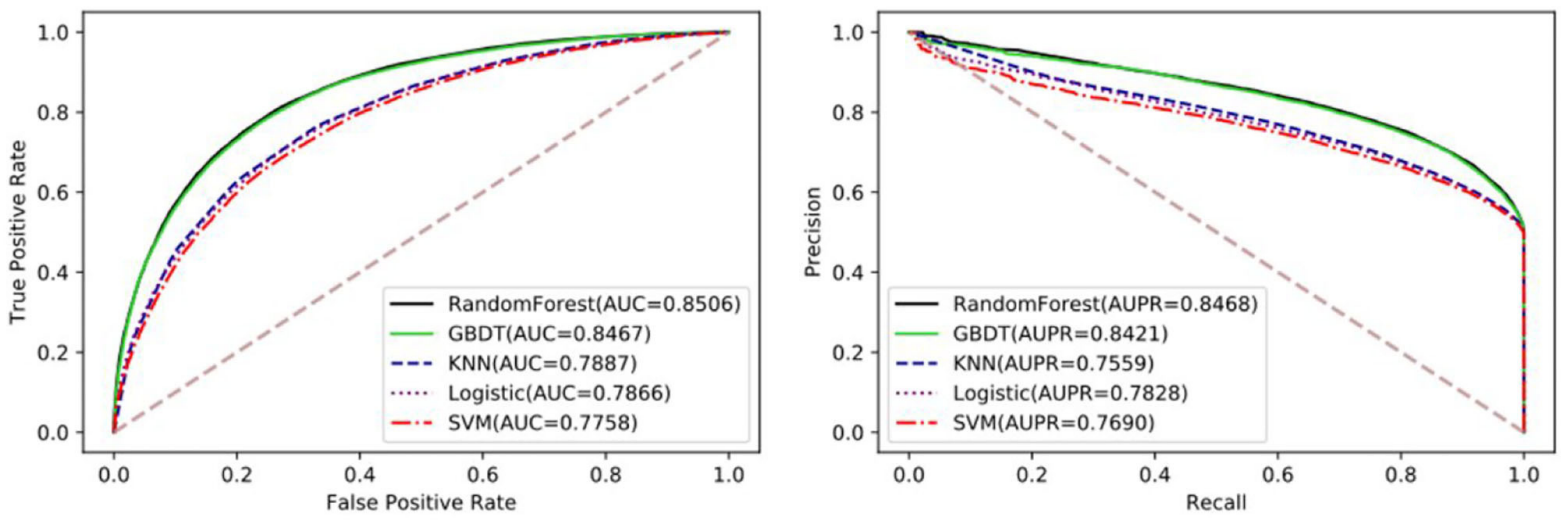
The performance comparison between Random Forest and GDBT, KNN, Logistic Regression, and SVM.

The performance of different machine learning classifiers in various fields may be different. In the dataset of this paper, we try to compare the differences of different machine learning algorithms, including SVM, Logistic Regression, KNN, Gradient Boosting Decision Tree (GDBT), and Random Forest Classifier. To better reflect the performance of each classifier on the dataset, they all go through parameter tuning and choose the optimal parameter for comparison. Here, we used the iterative method to find the optimal parameters. Detailed results of five-fold cross-validation based on different classifiers are shown in Table 3 and Figure 4.

**Table 3:** Comparison of different machine learning classifier under five-fold cross-validation.

**Classifier**	**Acc. (%)**	**Sen. (%)**	**Spec. (%)**	**Prec. (%)**	**MCC (%)**	**AUC (%)**
SVM	70.62 ± 0.85	71.71 ± 1.17	69.53 ± 1.61	70.20 ± 1.05	41.26 ± 1.69	77.58 ± 0.77
Logistic	71.48 ± 0.60	71.34 ± 0.78	71.61 ± 0.65	71.54 ± 0.59	42.95 ± 1.20	78.66 ± 0.56
KNN	69.13 ± 0.48	86.33 ± 0.34	51.92 ± 0.94	64.23 ± 0.45	40.74 ± 0.91	78.87 ± 0.60
GBDT	74.40 ± 0.43	60.90 ± 0.80	87.90 ± 0.78	83.44 ± 0.83	50.69 ± 0.91	84.67 ± 0.66
Random Forest	**76.86** **±** **0.49**	**73.86** **±** **0.79**	**79.86** **±** **0.61**	**78.57** **±** **0.53**	**53.82** **±** **0.97**	**85.06** **±** **0.49**

### Comparison With Other Association Prediction Methods

**Figure 5:**
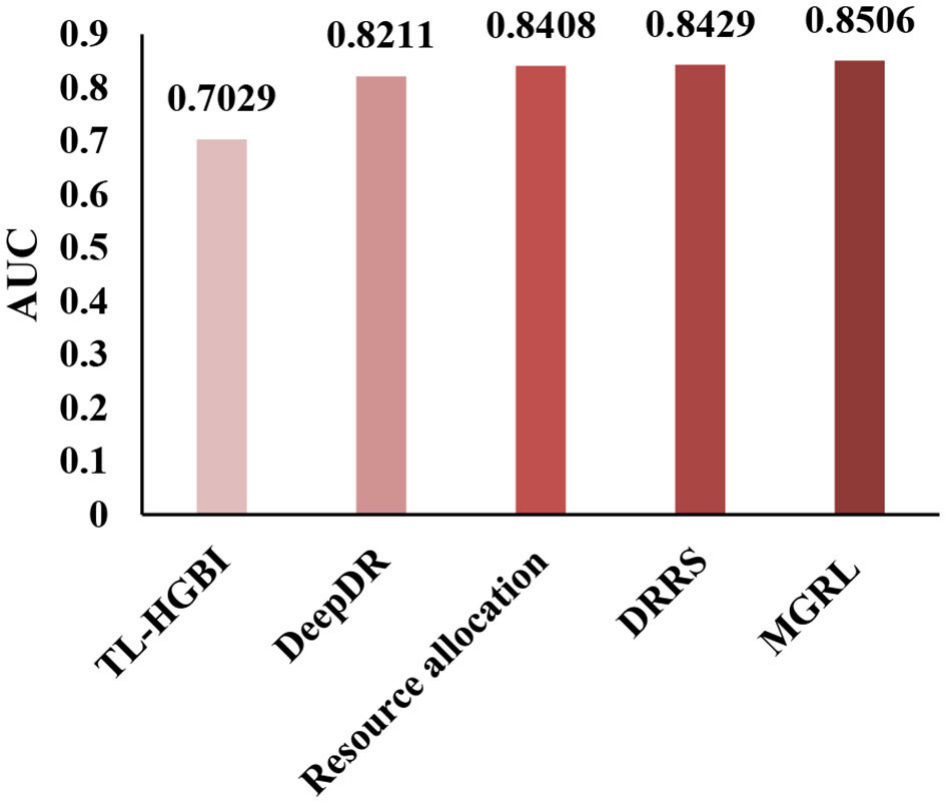
Under the CTD Dataset (contains 18,416 drug-disease associations between 269 drugs, and 598 diseases.), TL-HGBI, DeepDR, Resource allocation and DRRS were compared between the AUCs obtained under five-fold cross-validation.

To conduct a comprehensive analysis of MGRL, we demonstrate the superior performance of our method by comparing MGRL with the most advanced methods. Here, we compare MGRL with TL-HGBI (Wang et al., 2014), DeepDR (Zeng et al., 2019), the resource allocation method (Zhou et al., 2010), and DRRS (Luo et al., 2018) in the benchmark dataset by the five-fold cross-validation. The resource allocation method is a prediction method for predicting the problems of unobserved links in the bipartite graph. The results show that our method improves the AUC by 0.1477, 0.0295, 0.0098, and 0.0077 compared with other existing methods, and the results are shown in Figure 5. The proposed method constructed two kinds of node association graphs, trained the self-attribute of the node and the features of the association network, respectively, and significantly improved the prediction ability of the node.

### Case Study

To evaluate the performance of our model in practical application, we carried out case studies on five drugs Doxorubicin, Etoposide, Levodopa, Clonidine, and Ciprofloxacin. According to the model prediction, we obtained the predicted diseases and ranked them, and selected the top 10 candidate diseases, as shown in Table 4. Specifically, five drugs are selected from the benchmark dataset, and interactions between the drugs and the rest of the disease (excluding the original drug-disease associations) are established. These drug-disease interactions are used as the test set, and then MGNRL is used to make the prediction and get the corresponding score. Finally, the prior evidence of the drug and diseases was searched in the database and the literature. In addition, for drugs Doxorubicin and Etoposide, our model predicted that the top 10 candidates could be confirmed in CTD. For the remaining drugs, only one case of clonidine was unconfirmed, two cases of Levodopa were unconfirmed, and three ciprofloxacin cases were unconfirmed. The case studies demonstrated that our method can be used as an available tool for predicting the drug-disease associations. And it can help biomedical specialists to improve efficiency in clinical trials.

**Table 4:** The top 10 drug candidates of the five popular drugs supported by MGRL.

**Drug name**	**Rank**	**Disease name**	**Evidence**	**Rank**	**Disease name**	**Evidence**
Doxorubicin	1	Seizures	CTD	6	Hemolysis	CTD
	2	Headache	CTD	7	Drug eruptions	CTD
	3	Glioma	CTD	8	Cerebral hemorrhage	CTD
	4	Muscular diseases	CTD	9	Pancytopenia	CTD
	5	Drug hypersensitivity	CTD	10	Hyperbilirubinemia	CTD
Etoposide	1	Headache	CTD	6	Anemia, hemolytic	CTD
	2	Edema	CTD	7	Hypertension	CTD
	3	Thrombosis	CTD	8	Ovarian neoplasms	CTD
	4	Cholestasis	CTD	9	Ventricular dysfunction, left	CTD
	5	Exanthema	CTD	10	Carcinoma, hepatocellular	CTD
Levodopa	1	Depressive disorder	CTD	6	Ataxia	CTD
	2	Chemical and drug induced liver injury	CTD	7	Fever	CTD
	3	Inappropriate adh syndrome	CTD	8	Schizophrenia	CTD
	4	Tachycardia	CTD	9	Paresthesia	Unconfirmed
	5	Edema	CTD	10	Mood disorders	Unconfirmed
Clonidine	1	Headache	Unconfirmed	6	Long qt syndrome	CTD
	2	Memory disorders	CTD	7	Dystonia	CTD
	3	Chemical and drug induced liver injury	CTD	8	Nervous system diseases	CTD
	4	Bipolar disorder	CTD	9	Necrosis	CTD
	5	Cognition disorders	CTD	10	Psychotic disorders	CTD
Ciprofloxacin	1	Muscle weakness	CTD	6	Substance withdrawal syndrome	CTD
	2	Arrhythmias, cardiac	Unconfirmed	7	Hyperalgesia	CTD
	3	Necrosis	CTD	8	Tachycardia	CTD
	4	Liver diseases	CTD	9	Gastrointestinal diseases	CTD
	5	Sleep initiation and maintenance disorders	Unconfirmed	10	Anaphylaxis	Unconfirmed

## Conclusion

The increasing cost and duration of new drug development make the repositioning of existing drugs using computational methods a significant focus of medical or biological research. In this paper, we proposed a novel method MGRL to predict potential drug-disease associations. The proposed MGRL model establishes a high-dimensional feature vector through the deep integration of two graph representations of drugs and diseases, to enhance the feature information of nodes. The two kinds of graph feature vectors are spliced to get the final input feature vectors. In particular, the attributes of nodes are used, and perform further in-depth training through the graph convolutional neural network to improve the local characteristics of nodes. Experiments show that MGRL can achieve high-precision prediction of unobserved drug-disease associations, which is significantly better than other advanced methods. In future work, we will build a more complex drug-disease interactions network to mine more characteristic information and further improve the predictive ability of our model.

## Data Availability Statement

The datasets presented in this study can be found in online repositories. The names of the repository/repositories and accession number(s) can be found in the article/supplementary material.

## Author Contributions

B-WZ and Z-HY considered the algorithm, arranged the dataset, and performed the analyses. LWo, PZ, H-YL, and LWa wrote the manuscript. All authors read and approved the final manuscript.

## Conflict of Interest

The authors declare that the research was conducted in the absence of any commercial or financial relationships that could be construed as a potential conflict of interest. The reviewer WZ declared a shared affiliation with one of the authors PZ, to the handling editor at time of review.
